# *Lactobacillus plantarum* ZJUFB2 Prevents High Fat Diet-Induced Insulin Resistance in Association With Modulation of the Gut Microbiota

**DOI:** 10.3389/fnut.2021.754222

**Published:** 2021-10-14

**Authors:** Hao Zhong, Jian Wang, Abdullah, Muhammad Adnan Hafeez, Rongfa Guan, Fengqin Feng

**Affiliations:** ^1^College of Food Science and Technology, Zhejiang University of Technology, Hangzhou, China; ^2^Guangdong Provincial Key Laboratory of Nutraceuticals and Functional Foods, College of Food Science, South China Agricultural University, Guangzhou, China; ^3^National Institute of Food Science and Technology, University of Agriculture, Faisalabad, Pakistan; ^4^College of Biosystems Engineering and Food Science, Zhejiang University, Hangzhou, China

**Keywords:** *Lactobacillus plantarum*, metabolic syndrome, insulin resistance, gut microbiota, bile acids

## Abstract

*Lactobacillus plantarum* ZJUFB2 is a novel probiotic isolate derived from Chinese traditional sourdough that possesses promising probiotics properties. This study aimed to investigate the effects of *L. plantarum* ZJUFB2 (B2) on insulin sensitivity using mice fed on a high-fat diet (HFD) as well as to explore the involved mechanisms. Purposely, male C57BL/6 mice continuously received an intervention of B2 (~10^9^ CFU/day) for 16 weeks. The results showed that B2 treatment remarkably ameliorated insulin resistance and hyperglycemia in HFD-fed mice. The B2 intervention significantly decreased the hepatic lipid accumulation, serum low-density lipoproteins cholesterol, and lipopolysaccharides, and regulated the bile acids levels as well as liver mRNA expression involved in lipid metabolism. Moreover, the B2 intervention significantly changed the gut microbiota, specifically, showed a lower abundance of obesity-related and inflammation-associated microbes, e.g., *Ruminococcus* and *Mogibacteriaceae*. Furthermore, it exhibited a higher abundance of short-chain fatty acids and bile salt hydrolas-producing bacteria, such as *Bifidobacterium* and *F16* compared with the HFD group. The findings of this study suggested that B2 is a novel probiotic, effective in preventing insulin resistance by improving the gut microbiota and bile acids.

## Key Points

- *L. plantarum* ZJUFB2 alleviated insulin resistance and fat accumulation in the liver.
- B2 significantly altered the gut microbiota and their metabolites, e.g., SCFAs, bile acids.
- Roles of the axis “gut microbiota—bile acids—liver” associated with the B2 were investigated.

## Introduction

Type 2 diabetes mellitus is a public pandemic that remains a challenging health issue of the 21st century which leads to the onset of plenty of health risks and economic burdens (1). The disease owes to the relative lack of insulin mainly caused by insulin resistance (IR) in fuel storage organs, e.g., skeletal muscles, liver, and white adipose tissues (2). Despite increasing knowledge of pathogenesis and management of IR, the global prevalence and incidence of type 2 diabetes mellitus (T2DM) is continuously growing (3). Many studies indicated that systemic inflammation and fat accumulation were the key factors in triggering abnormal cellular responses to insulin, resulting in IR (4, 5). In this regard, regulating the pro-inflammatory cytokines expression and fat accumulation are the challenging concerns in preventing obesity-related inflammation and subsequently insulin sensitivity.

The intestinal ecosystem comprising a complex microbial community. In the case of any imbalance in the gut microbiota, they can affect the normal metabolic pathways which lead to immune dysfunctions (6, 7). It has been reported that gut microorganisms play a crucial role in regulating the host glucose homeostasis (8, 9). Many studies have indicated that intestinal microflora dysbiosis may trigger the onset of diabetes and obesity-related issues (10). In a related study, sterile rodents showed resistance to high-fat diet (HFD) induced obesity, indicating that the gut microbiota may influence the mammalian adiposity through regulation of the metabolic pathways that control the bioenergetics (11). The study of Amar et al. (12) reported that the commensal gut microbiota translocation resulting in the early development of T2DM was associated with an HFD (12). In addition, the change in the intestinal microbiota composition and diversity also contributes to the development of T2DM and obesity. For instance, an increase in the growth of pathogens may cause disruption in the gut barrier which increases gut permeability, resulting in hyperglycemia (IR), inflammation, and obesity-related issues (13). The abundance of certain specific bacteria, such as *Oscillospira* and *Lactococcus*, were closely related to the inflammatory cytokines and obesity of the body (14). The level of *Bifidobacterium* was positively correlated with expanded glucose tolerance and gut barrier in mice (15, 16). Although the mechanism regarding the interaction of the gut microbiota with the host is still not clear, few studies have demonstrated that a liver-bile acids (BAs)-gut microbiome metabolic axis drives significant modifications of BAs and microbiota composition capable of triggering metabolic disorders (17). The small heterodimer partner (SHP), which controls the metabolic pathways, is involved in the glycolipids homeostasis and hepatic BAs production through the transcriptional repression of several other nuclear receptors (18).

Probiotics have been widely used to combat IR since it is an effective and direct intervention for the gut microbiota and BAs production. In recent years, studies demonstrated that *Lactobacillus plantarum* strains were found effective against diarrhea, irritable bowel syndrome, metabolic syndrome, and especially hyperglycemia (19–21). Recently, clinical trials indicated that *L. plantarum* intervention prevented/delayed diabetes occurrence by regulating blood glucose, improving insulin sensitivity, and minimizing oxidative stress (22, 23). Therefore, it is hypothesized that *L. plantarum* (specific strain) may possess antidiabetic potentials and beneficial effects in the management of T2DM.

In this study, a novel isolate *L. plantarum* ZJUFB2 (B2) derived from sourdough, a Chinese traditional breadmaking starter, which possesses excellent *in vitro* probiotics properties was evaluated for its anti-diabetic potentials using a mice model, in which IR was induced by HFD. Afterward, HFD-fed C57BL/6 mice were orally administered with B2 for 16 weeks and investigated with the following objectives, including (i) glucose and lipid metabolism profile, (ii) inflammatory cytokines expression, total bile acids, and the intestinal microflora, and (iii) liver mRNA levels involved in glycolipids metabolism.

## Materials and Methods

### Acquiring of the *L. plantarum* ZJUFB2

*Lactobacillus plantarum* ZJUFB2 (stored at China center for type culture collection, CCTCC M2020126) was screened from Chinese traditional fermented food (sourdough). The B2 tested sample was manufactured according to the reported guidelines (24). The concentration of the lyophilized B2 sample was ~10^11^ colony forming units per gram (CFU/g).

### Animal Experiment Design

Male C57BL/6 mice (*n* = 36) of 6-weeks were acquired from the Shanghai SLAC Laboratory Animal Co., Ltd. (Shanghai, China), housed at 60% relative humidity and 25°C, and provided with free access to water and diet. After a week of acclimatization, the mice were divided into three groups (*n* = 12, 04/cage). The mice of different groups were given a normal chow diet (NCD, 10% calories from fat, MD17111, Jiangsu Medicine Ltd., Supplementary Table 1), HFD (45% calories from fat, MD12032, Jiangsu Medicine Ltd., Supplementary Table 1), and an oral intervention of 0.2 ml of saline containing ~10^9^ CFU bacteria daily for a period of 16 weeks: (i) NCD group (NCD, saline), (ii) HFD group (HFD, saline), and (iii) B2 group (HFD, B2), respectively. Afterward, mice were fasted up to 12 h and euthanized using pentobarbital sodium (100 mg/kg, *i.v*.) to collect the samples. Serum samples were obtained by centrifugation of blood (3,000 × *g*, 15 min). The samples including mice livers, inguinal white adipose tissue (iWAT), epididymal white adipose tissue (eWAT), and brown adipose tissue (BAT) were obtained, weighed, and stored at −80°C.

### Glucose Homeostasis

After a 15-weeks feeding, male C57BL/6 mice were fasted (12 h) followed by an intraperitoneal glucose tolerance test (GTT) was conducted after glucose injection (2 g/kg of the bodyweight). Blood glucose was determined with an interval of 0, 30, 60, 90, and 120 min using a glucometer (Accu-Check glucometer, Roche, Mannheim, Germany). Fasting serum insulin and serum glucose levels were measured by ELISA and glucose oxidase assay kits (Jiyinmei, Wuhan, China, and Jiancheng Bioengineering Institute, Nanjing, China). Furthermore, the insulin resistance in terms of homeostasis model assessment was determined using this equation:

Homeostasis model assessment of insulin resistance (HOMA-IR) = Fasting serum glucose (mM) × Fasting serum insulin (mU/L)/22.5.

### Biochemical Determination

The serum biochemical parameters including low-density lipoproteins cholesterol (LDL-C), high-density lipoproteins cholesterol (HDL-C), total cholesterol (T-CHO), triacylglycerols (TG), total bile acids (TBAs), and hepatic glutathione peroxidase enzyme (GSH-Px), total superoxide dismutase enzyme (SOD), and catalase enzyme (CAT) were measured using corresponding kits (mention names) (Jiancheng Bioengineering Institute, Nanjing, China). Lipopolysaccharide (LPS) content was detected by a ToxinSensor Chromogenic Limulus Amebocyte Lysate (LAL) (Endotoxin Assay Kit, GenScript, Piscataway, New Jersey, USA).

Serum lipopolysaccharide-binding protein (LBP), leptin (LEP), adiponectin (ADP), glucagon-like peptide-1 (GLP-1), peptide YY (PYY), hemoglobin A1c (HbA1c), tumor necrosis factor-alpha (TNF-α), interleukin 10 (IL-10), and interleukin 6 (IL-6) levels were determined using commercial ELISA kits (Jiyinmei, Wuhan, China).

### Histological Analysis

The hematoxylin and eosin (HandE) staining procedures and analysis were performed following the procedures of Zhong et al. (25). To determine the size of the adipocytes, 10 different random visual fields were computed for each HandE staining image.

### Q-PCR

All the reagents for extracting total RNA, synthesizing cDNA, and Quantitative PCR (qPCR) procedures were carried out using commercial kits (Vazyme Corp., Nanjing, China). The PCR program and calculation process were conducted following our previous study (25). The primers of sterol regulatory element-binding protein 1c (SREBP-1c); fatty acid synthase (FAS); peroxisome proliferator-activated receptors (PPARγ and PPARα), cholesterol 7α-hydroxylase (CYP7A1), and SHP used for performing RT-qPCR were listed in Supplementary Table 2.

### 16S rRNA Analysis of the Gut Microbiota

For gut microbiota analysis, the procedure was carried out according to our study (25). Briefly, fresh fecal samples (*ca*. 30 mg, *n* = 8) on 16 weeks were collected and immediately frozen using dry ice and stored at −80°C. Total DNA was extracted from fecal samples using a QIAamp DNA Stool Mini Kit (Qiagen, Venlo, Netherlands) according to the instructions of the manufacturer. The V3-V4 hypervariable regions of bacteria 16S rRNA gene were amplified with primers 338F (5′- ACTCCTACGGGAGGCAGCAG-3′) and 806R (5′-GGACTACHVGGGTWTCTAAT-3′) by thermocycler PCR system (GeneAmp 9700, ABI, USA). Library quality was checked using a Thermo NanoDrop 2000 ultraviolet microspectrophotometer, and 2% agarose gel electrophoresis as well as Qubit for library quantification (Thermo Scientific, USA). Illumina MiSeq platform (Majorbio Bio-pharm Technology Co. Ltd., Shanghai, China) was used to sequence. The analysis protocol was performed following our previous work (25). Sequences with 97% similarity were clustered into operational taxonomic units (OTUs) using UPARSE (V7.0.1001) (http://drive5.com/uparse/). A representative sequence was picked for each OTU and the Greengenes reference database was used to annotate taxonomic information for each representative sequence. The OTU absolute abundance table was extracted from the pipeline and converted to relative abundances by normalizing to total OTU clustering for analyzing the composition of gut microbiota by Quantitative Insights into Microbial Ecology software (QIIME) (http://qiime.org). In order to compare diversity, the OTU table was rarified to calculate the metrics as Chao1, Observed species, and Shannon index. 3D principal coordinate analysis (PCoA) plot was constructed for the evaluation of the dissimilarity and the community composition between samples from weighted UniFrac distances. Linear discriminant analysis (LDA) effect size (LEfSe) was performed to identify the difference between groups. The correlation analysis between the intestinal bacteria and significant biomarkers in mice was calculated by the R software (R version 3.6.3) (Free Software Foundation, USA). Raw Illumina MiSeq reads were publicly accessible in the NCBI Sequence Read Archive (SRA) database with accession number PRJNA723728.

### SCFAs Analysis

The short-chain fatty acids (SCFAs) analysis was performed following the protocol of our previous study (25). The results of SCFAs were shown in μmol/g of a fecal sample.

### Statistical Analysis

The obtained data were analyzed statistically to determine the level of significance using one-way ANOVA along with a *post-hoc* Tukey's test. The statistical analysis was conducted through SPSS 22 (SPSS Inc., Chicago, IL, USA). GraphPad Prism 6 (GraphPad Software Inc., San Diego, CA, USA) was used to shape the experimental data, and the data shown in the manuscript indicating mean ± SE (*n* = 8–12). Means with the same letters in a table or a figure differ non-significantly (*P* > 0.05).

## Results

### B2 Prevented Hepatic Lipid Accumulation

**Figure 1:**
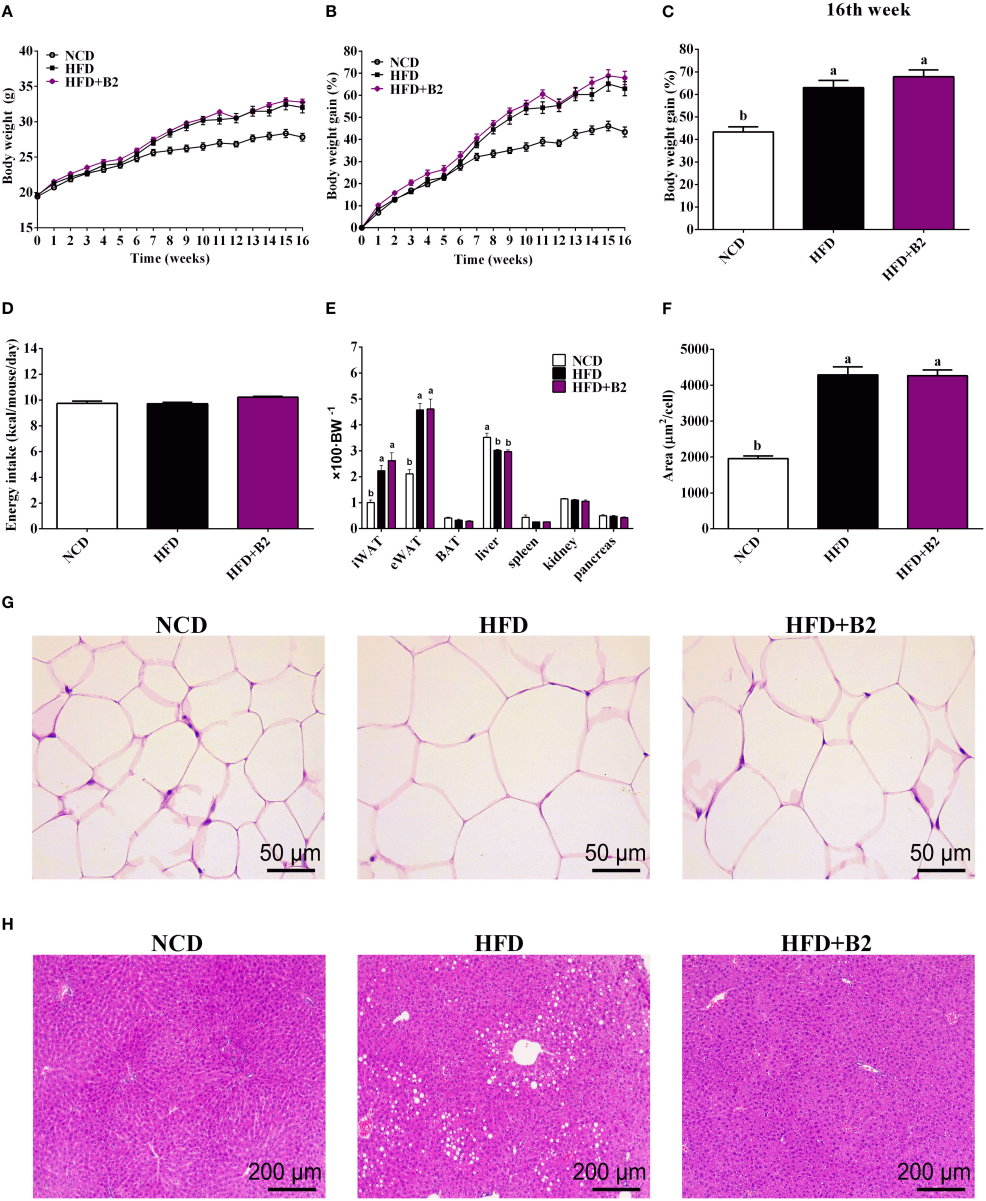
*Lactobacillus plantarum* ZJUFB2 (B2) administration did not affect the body weight gain and eWAT, but suppressed lipid accumulation of liver in HFD-induced mice. **(A)** body weight of mice during 16 weeks (*n* = 12); **(B)** body weight gain changes (*n* = 12); **(C)** weight percentage (*n* = 12); **(D)** average daily energy intakes (*n* = 12); **(E)** the weight percentage of organs (*n* = 12); **(F)** adipocyte size of eWAT (*n* = 6); **(G)** HandE staining of eWAT (*n* = 6); **(H)** HandE staining of liver tissues (*n* = 6).

After 8 weeks, the body weight and total body weight gain (TBWG) of HFD and B2 groups were increased significantly (Figures 1A,B) as compared with the NCD group. At the end of the experiment, mice fed on B2 showed a relatively higher percentage of body weight gain compared with the NCD group, but no significant difference was observed in comparison to the HFD group (Figure 1C). However, no significant difference was observed in daily energy intakes among the groups, indicating that an HFD did not change energy intakes (Figure 1D). Similarly, there was no significance found in the weight of liver and fat tissues, and the size of eWAT between HFD and B2 groups (Figures 1E–G). Intriguingly, Figure 1H showed that the number of multi-locular lipid droplets in the livers of the B2 group were lower than in the HFD group. Based on the findings of this study, B2 exhibited strong inhibitory effects on the lipid formation in the liver of HFD-fed mice without disturbing their body weight.

### B2 Hypolipidemic and Insulin-Sensitivity Potentials

**Figure 2:**
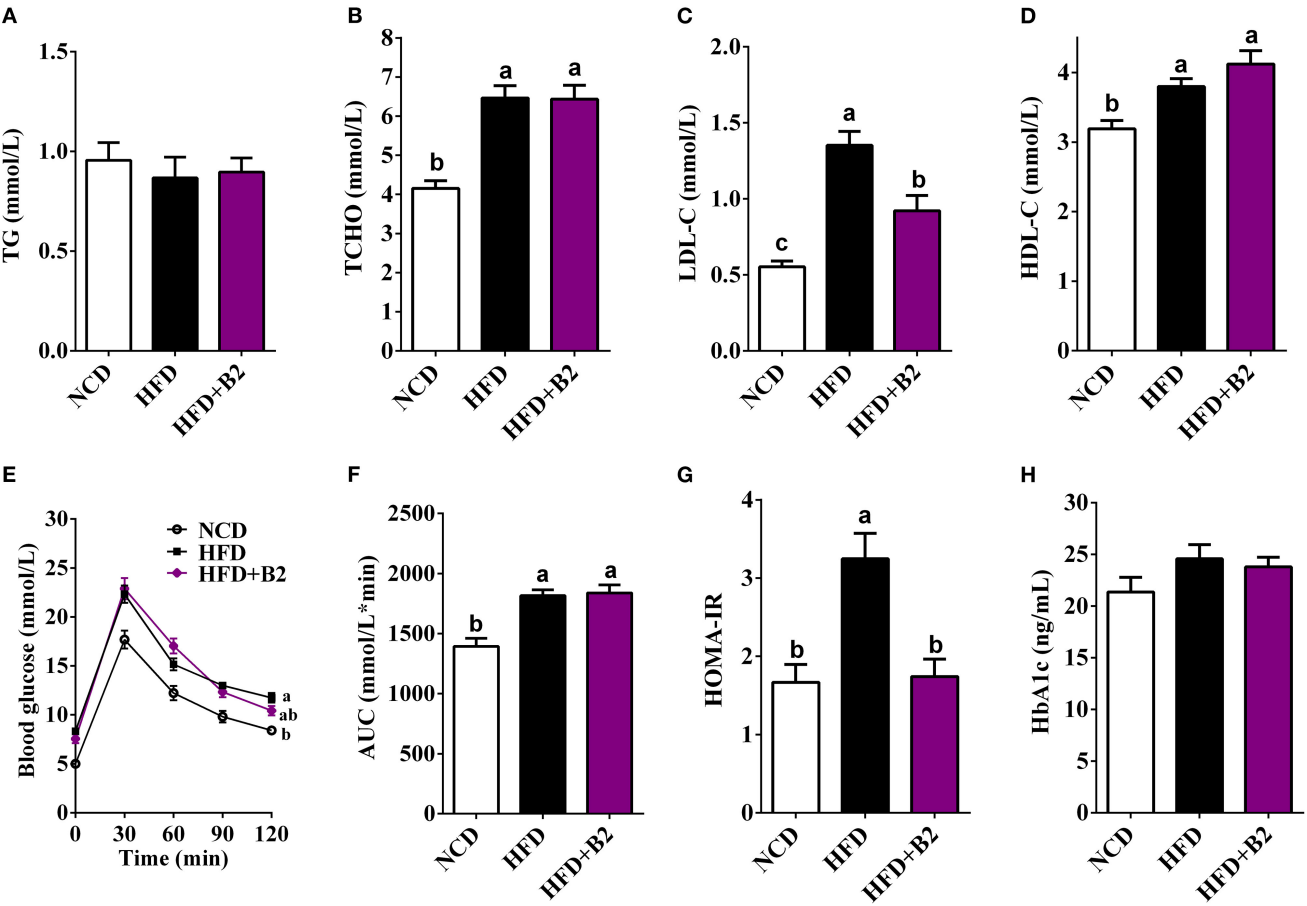
Effects of B2 on serum lipid profiles and glucose homeostasis. **(A)** TG; **(B)** TCHO; **(C)** LDL-C; **(D)** HDL-C; **(E)** glucose tolerance test (GTT); **(F)** area under curve (AUC) from GTT; **(G)** HOMA-IR; **(H)** HbA1c. Data were shown as mean ± SE (*n* = 8).

High-fat diet group did not exhibit any effects on the serum TG (Figure 2A), but TCHO contents increased (from 4.16 to 6.4 mmol/L) in HFD-fed mice compared with the NCD group (Figure 2B). However, mice who received B2 intervention exhibited a decline in LDL-C (Figure 2C). Likewise, the B2 group showed a slight increase in the HDL-C in comparison to the NCD group (Figure 2D). In the GTT result, Figure 2E demonstrated that less glucose tolerance was observed in HFD-fed mice. The B2 intervention did not show any improvement in the glucose tolerance in comparison with the HFD group (Figure 2F). An HFD significantly elevated fasting serum glucose in mice but fasting serum insulin and HbA1c of NCD- and HFD-fed mice showed no significant differences (Table 1; Figure 2H). However, the data of HOMA-IR (from 3.25 to 1.74) and serum fasting glucose (from 6.96 to 3.7 mmol/L) showed that B2 intervention significantly decreased insulin resistance and hyperglycemia (Figure 2G; Table 1).

**Table 1:** Fasting serum glucose (GLU) level and insulin (INS) level.

	**NCD**	**HFD**	**HFD+B2**
GLU (mmol/L)	3.92 ± 0.38^b^	6.96 ± 0.45^a^	3.70 ± 0.42^b^
INS (mU/L)	9.57 ± 0.80	10.43 ± 0.66	10.54 ± 0.34

*The significant difference between groups (n = 12) was represented by different letters (P < 0.05)*.

### B2 Improved Serum Hormone and Cytokines

**Figure 3:**
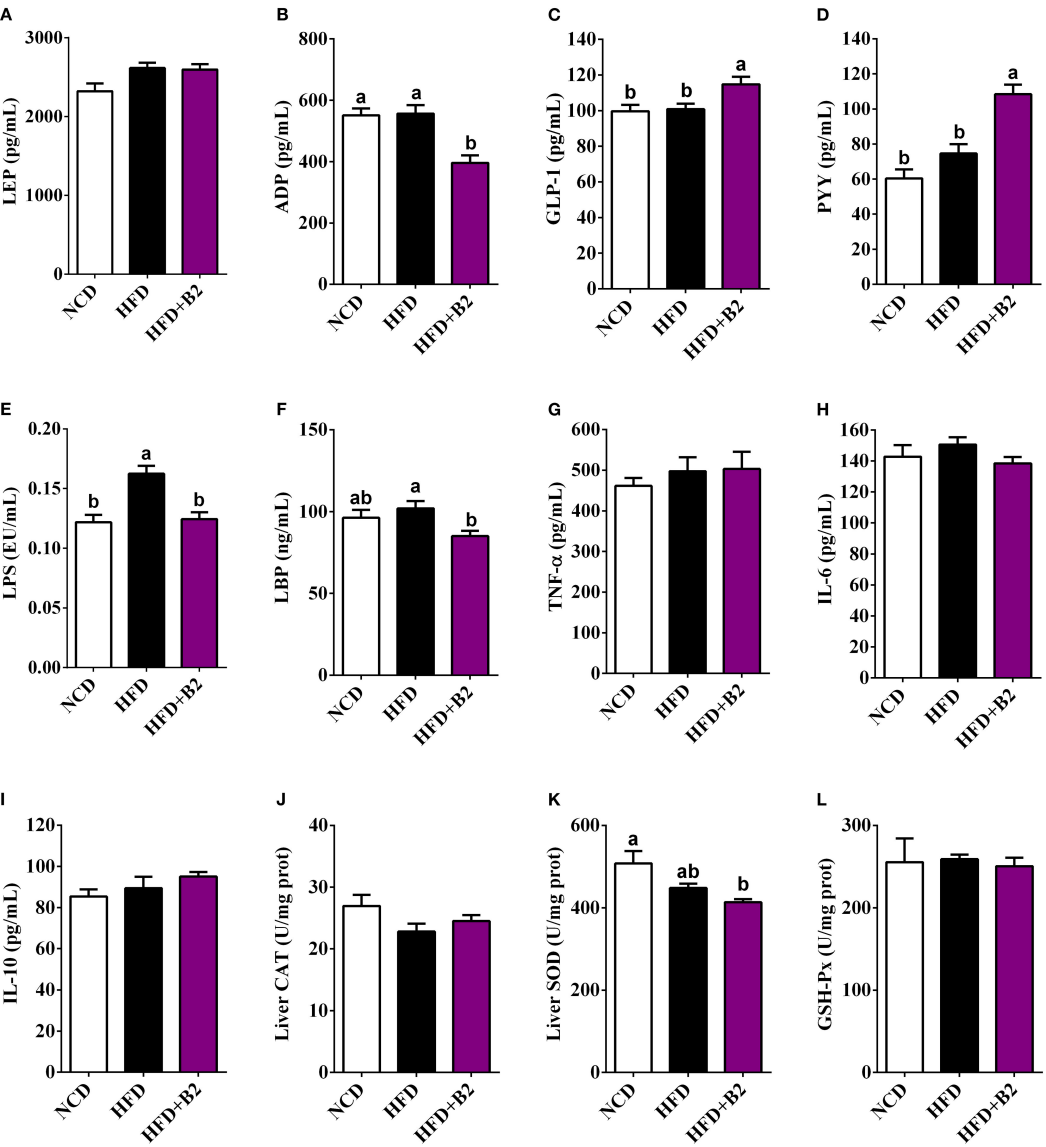
Effects of B2 on serum hormones, cytokines, and liver oxidative stress. **(A)** LEP; **(B)** ADP; **(C)** GLP-1; **(D)** PYY; **(E)** LPS; **(F)** LBP; **(G)** TNF-α; **(H)** IL-6; **(I)** IL-10; **(J)** liver CAT; **(K)** liver SOD; **(L)** liver GSH-Px. Data were shown as mean ± SE (*n* = 8).

**Figure 4:**
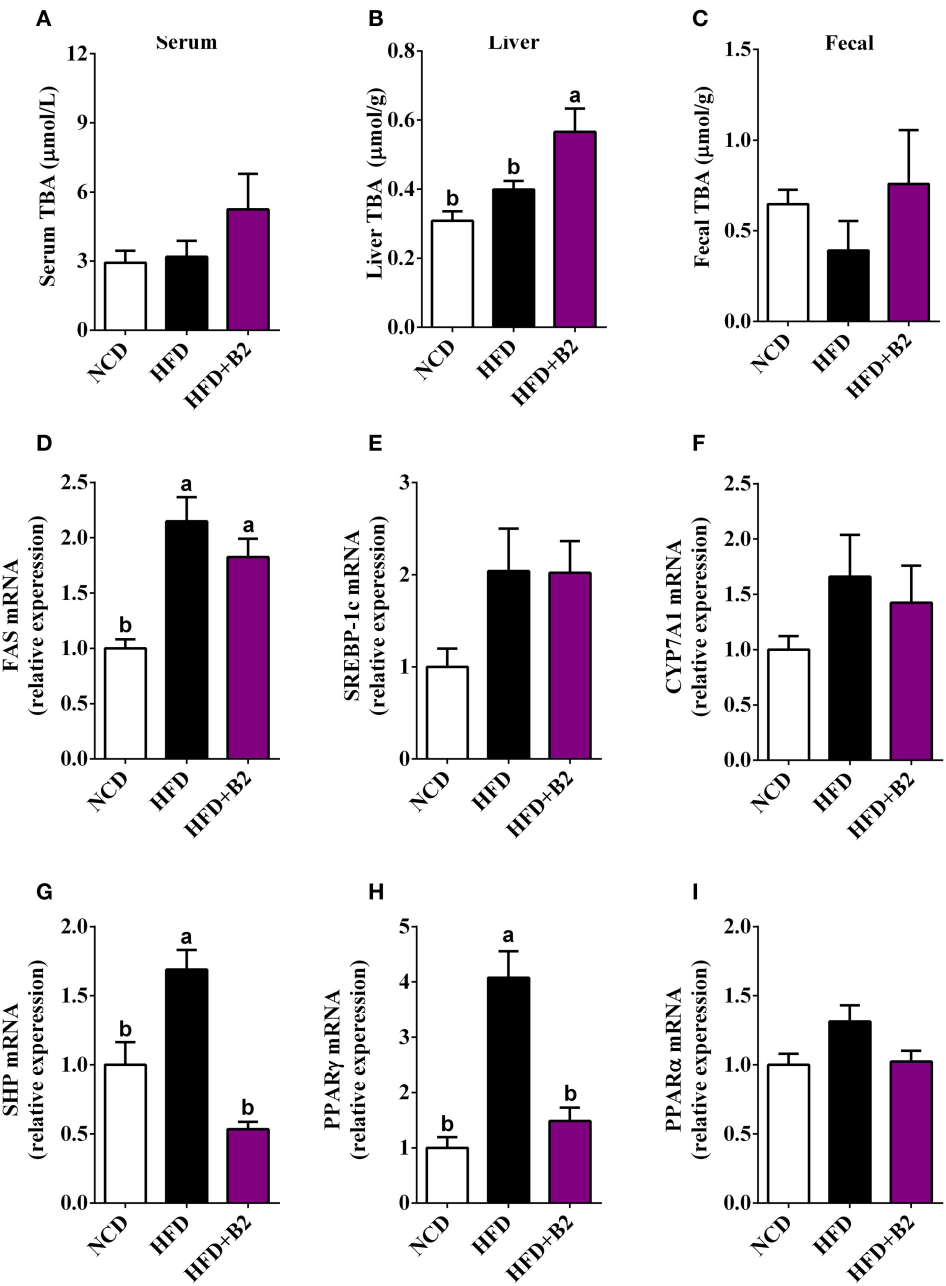
Effects of B2 on total bile acids (TBA) and liver gene expression in HFD-fed mice (*n* = 8). **(A)** serum TBA; **(B)** liver TBA; **(C)** fecal TBA; **(D)** relative expression of mRNA of *FAS*; **(E)** *SREBP-1c*; **(F)** *CYP7A1*; **(G)** *SHP*; **(H)** *PPAR*γ; **(I)** *PPAR*α.

To evaluate changes in the hormone levels with B2 treatment, the serum LEP, ADP, PYY, and GLP-1 levels were determined. Figures 3A,B, indicate that the mice group fed with B2 showed no significant change in the LEP levels compared with the HFD group, but a decrease was observed in the ADP levels. B2 supplementation in HFD-fed mice significantly increased the serum GLP-1 and PYY levels compared with the NCD and HFD groups (Figures 3C,D). The serum LPS levels in the HFD group were significantly increased compared with the NCD group (Figure 3E). Overall, B2 intervention significantly improved HFD-induced increased levels of LPS and LBP (Figures 4E,F). Although there were some positive modifications in TNF-α, IL-6, and IL-10 levels after B2 administration, but no significant differences were observed (*P* > 0.05) (Figures 3G–I). Mice fed with B2 exhibited a slightly higher content of IL-10 (Figure 3I) and a lower concentration of IL-6 (Figure 3H) than that of the HFD group. Figures 3J–L showed that there were no significant differences in liver antioxidant enzymes between HFD and B2 groups.

### B2 Modulated Bile Acids Metabolism and Lipid Metabolism-Related Gene Expression

To investigate that B2 intervention would alter the gut microbiota and lipid metabolism, the contents of bile acids and lipometabolism-related genes expression levels were determined. The total bile acids data showed a significant increase in the liver tissues with B2 intervention compared with the NCD and HFD groups, while there was a slight increase in fecal and serum samples (Figures 4A–C). The results of RT-qPCR showed that the mRNA expression levels of *SREBP-1c, CYP7A1*, and *PPAR*α have not been significantly altered after B2 intervention (Figures 4E,F,I). The expressions of the *FAS* were upregulated in HFD and B2 groups as compared with the NCD group (Figure 4D). The mRNA expression levels of *PPAR*γ and *SHP* were found lower in the B2 group than HFD group (Figures 4G,H).

### B2 Intervention Altered the Gut Microorganism

**Figure 5:**
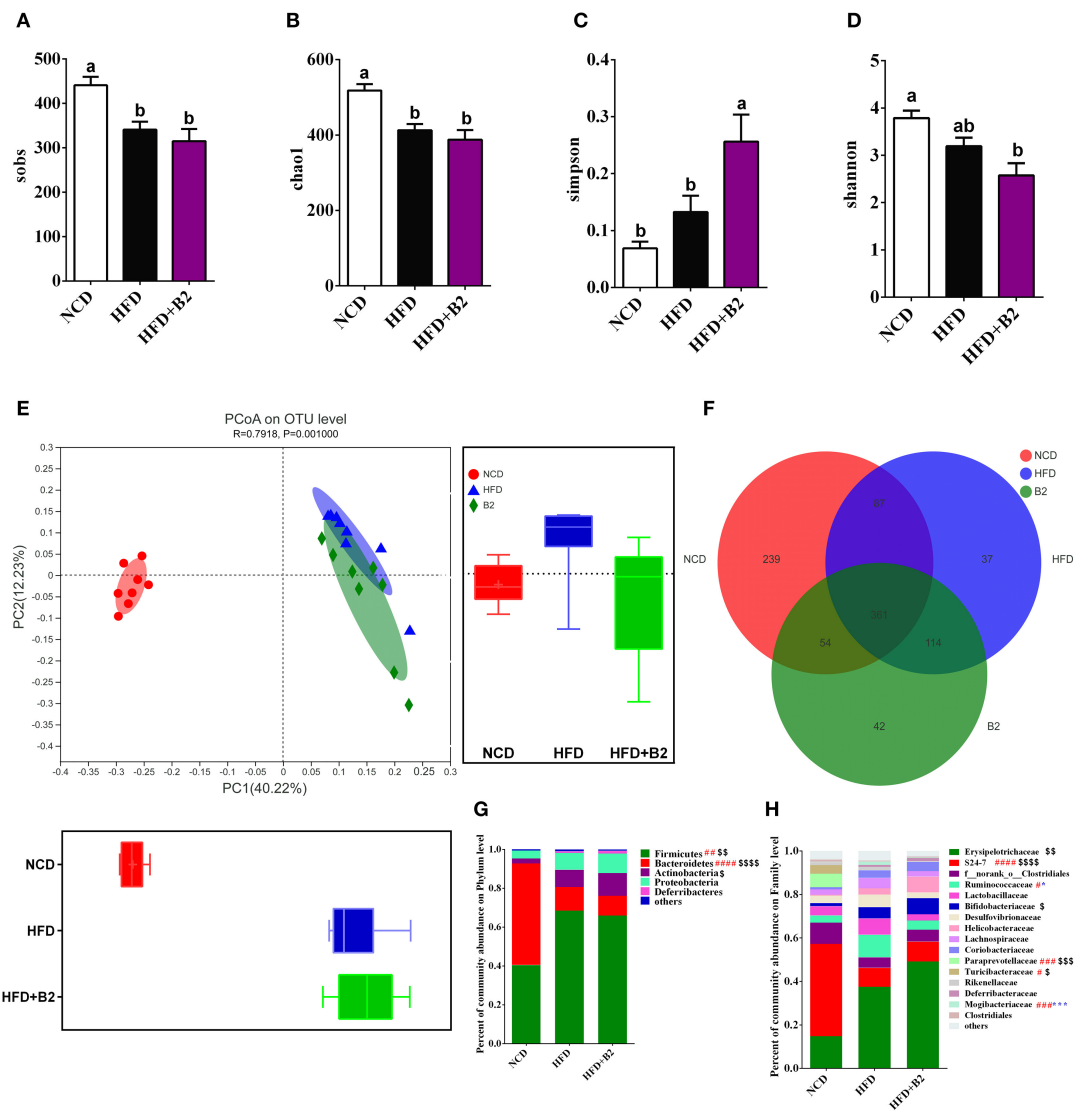
Effects of B2 on diversity of the gut microbiota. **(A)** Sobs; **(B)** Chao1; **(C)** Simpson; **(D)** Shannon; **(E)** PCoA plot (weighted UniFrac distances); **(F)** OTUs Venn Diagram; **(G)** Phylum level; **(H)** Family level. ^#^*P* < 0.05 NCD vs. HFD, ^$^*P* < 0.05 NCD vs. HFD+B2, and *P < 0.05 HFD vs. HFD+B2 (*n* = 8).

The α-diversity (diversity parameter of Sobs and Chao1) of the gut microbiota was significantly decreased in the HFD-fed group compared with the NCD group (Figures 5A,B). However, the results for Simpson and Shannon diversities showed a decrease in the B2 group (Figures 5C,D) compared with the HFD group, indicating that the B2 intervention slightly decreased the gut microbial α-diversity. To investigate the difference in the microbial structure among the different groups, a PCoA was performed to calculate the similarity of community composition (Figure 5E). HFD and B2 groups formed a peculiar composition of microflora independently from the NCD group. Moreover, the B2 group had a separated cluster as compared with the HFD group. Figure 5F showed that the HFD and B2 groups shared 448 and 415 common OTUs with the NCD group, respectively. The classification abundance analysis showed that the HFD group had a higher relative abundance of *Firmicutes* and a lower *Bacteroidetes* in comparison with NCD-fed mice. After B2 supplementation, the abundances of *Firmicutes* and *Bacteroidetes* showed a decline, and *Actinobacteria* and *Proteobacteria* increased compared with the HFD group (*P* > 0.05, Figure 5G). The results of community abundance on Family level showed that B2 had a lower abundance of *Ruminococcaceae* (*P* < 0.05), *Lactobacillaceae, Desulfovibrionaceae, Lachnospiraceae*, and *Mogibacteriaceae* (*P* < 0.05), and a higher abundance of *Bifidobacteriaceae, Helicobacteraceae*, and *Deferribacteraceae* in comparison with HFD group (Figure 5H).

**Figure 6:**
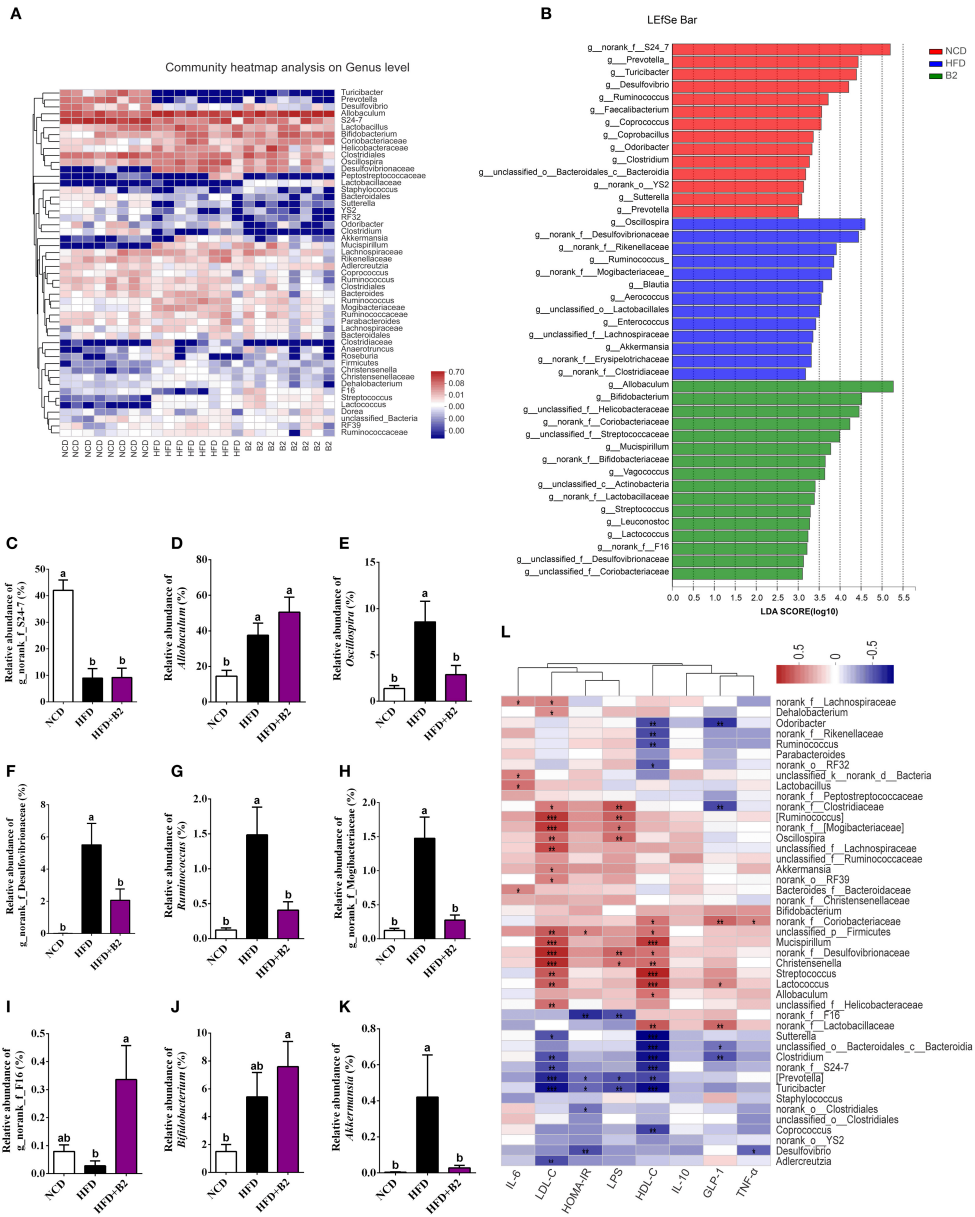
Effects of B2 on the gut bacteria composition. **(A)** Heatmap analysis on genus level (the relative abundance of OTUs >0.25%); **(B)** The LEfSe analysis (LDA > 3); **(C)** The relative abundance of *g_norank*_f_S24-7; **(D)** *Allobaculum*; **(E)** *Oscillospira*; **(F)** *g_norank*_*f_Desulfovibrionaceae*; **(G)** *Ruminococcus*; **(H)** *g_norank*_*f_Mogibacteriaceae*; **(I)** *g_norank*_f_F16; **(J)** *Bifidobacterium*; **(K)** *Akkermansia*; **(L)** Correlations between intestinal microflora and biochemical indicators. In **(L)**, *P* < 0.001 marked with “^***^”, *P* < 0.01 marked with “^**^”, *P* < 0.05 marked with “^*^” (*n* = 8).

The gut microbiota of experimental mice was mainly composed of 50 genera (Figure 6A). The consumption of an HFD significantly increased the relative abundance of *Allobaculum, Oscillospira, Bifidobacterium, norank_f__Desulfovibrionaceae, unclassified_f__Helicobacteraceae, Ruminococcus, norank_f_Mogibacteriaceae, Mucispirillum, Streptococcus, unclassified_f__Lachnospiraceae, unclassified_f__Ruminococcaceae, Lactococcus*, and *Dehalobacterium*, and significantly decreased the relative abundance of *norank_f__S24-7, Prevotella, Turicibacter, Desulfovibrio, Clostridium, unclassified_o__Bacteroidales_c__Bacteroidia, Sutterella, Coprococcus, Adlercreutzia*, and *norank_o__YS2*. To further examine the specific effects of B2 on the gut microbiota in HFD-fed mice, a LEfSe analysis was used to find the distinct bacteria in each group (Figure 6B). Figures 6C–K showed the modifications of the abundance of the characterized genera of bacteria base on the LEfSe report. The predominant genus in the NCD group was *g_norank_f__S24-7* and in the HFD-fed mice was *Allobaculum* (Figures 6C,D). The administration of B2 dramatically decreased the obese-type gut microbiota (*Oscillospira, norank_f__Desulfovibrionaceae, Ruminococcus*, and *norank_f_Mogibacteriaceae*) were significantly decreased after B2 intervention (Figures 6E–H), and the alteration in their abundance was in line with the modification in HOMA-IR. The relative abundance of probiotics *Bifidobacterium* was also explored (Figure 6J). B2 treatment significantly elevated its relative abundance (*P* < 0.05) in comparison with the HFD group. Moreover, *F16*, a biomarker in the B2 group, was remarkably increased after probiotic administration (Figure 6I), while *Akkermansia* was decreased (Figure 6K). Finally, Spearman's correlation analysis was applied to investigate the relationship between the intestinal microflora and biochemical profiles (Figure 6L). Microbes such as *F16* and *g_norank_f__Lactobacillaceae*, were found significantly enriched in the B2 group and were negatively correlated with HOMA-IR and LPS. In contrast, *Oscillospira, Lactococcus*, and *Mucispirillum* had positive correlations with LDL-C and LPS.

### B2 Increased the Fecal SCFA Content

**Figure 7:**
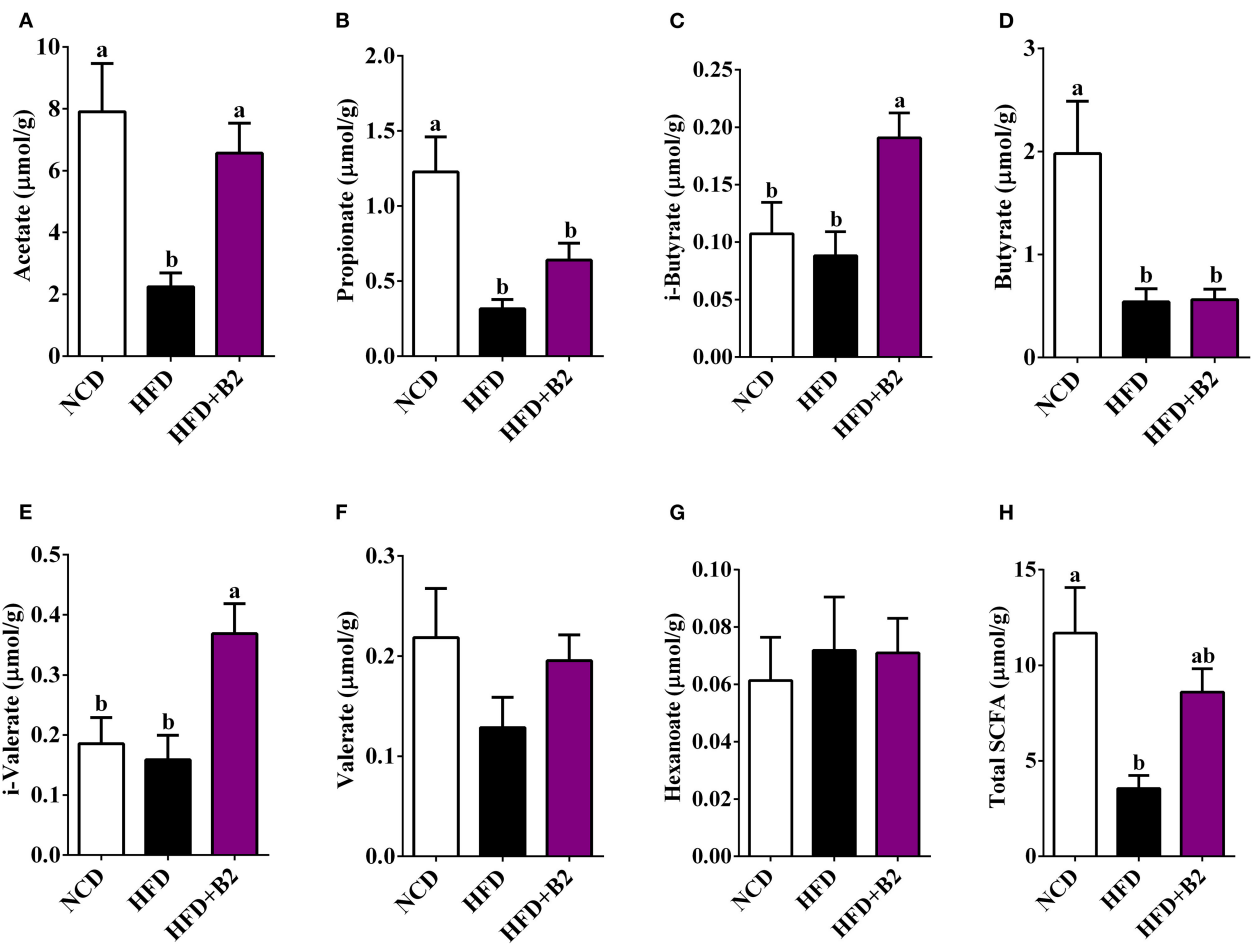
B2 intervention increased caecum content of SCFAs in HFD-fed mice. **(A)** Acetate; **(B)** Propionate; **(C)** i-Butyrate; **(D)** Butyrate; **(E)** i-Valerate; **(F)** Valerate; **(G)** Hexanoate; **(H)** Total SCFAs. Data were shown as mean ± SE (n = 8).

Figure 7 shows that B2 intervention significantly increased the total contents of fecal SCFAs as compared with the HFD group (Figure 7H). Specifically, B2 intervention significantly increased the levels of acetate, i-butyrate, and i-valerate in comparison with the HFD group (Figures 7A,C,E). Moreover, the B2 group showed a relatively higher amount of propionate and valerate than the HFD group (Figures 7B,F).

## Discussion

Studies have reported the hypoglycemic and weight-reducing potentials of *L. plantarum* in animal models, whereas the underlying mechanisms have not yet been thoroughly studied. Recently, some studies reported the potentials of the gut microbiota in glycolipids metabolism and related diseases (7, 26). The findings of this study inferred that B2 intervention not only prevented HFD-induced IR but also reduced inflammation by changing the gut microflora. In addition, this green intervention also plays an important role in regulating the genes associated with the glucose and lipid metabolism in the liver. Thus, the gut microbiota and bacteria-related biomarkers of mice fed on B2 were investigated to understand the possible mechanisms of hypoglycemia.

Generally, an HFD triggers the process of visceral fat mass, leading to metabolic dysfunction. In this case, B2 decreased lipid accumulation in the liver but did not impede HFD-induced body weight gain and lipid accumulation in white adipose tissues. Many reports have demonstrated that probiotics may have no potential in preventing obesity, but improving non-alcohol fatty liver disease and hyperlipidemia (27, 28). Similarly, B2 treatment ameliorated the high level of LDL-C in HFD-fed mice. Notably, in this study, B2 prevented hyperglycemia and insulin resistance, probably by improving the gut microbiota and inhibiting LPS synthesis. Therefore, B2 may play an important role in maintaining blood glucose and lipid homeostasis.

In addition, inflammation also plays a negative role in the occurrence and development of hyperglycemia. The increase of plasma LPS induced by an HFD has been found to trigger inflammation and IR (29, 30). In this study, HFD-fed mice exhibited a remarkably higher level of LPS. Correspondingly, the slightly higher serum levels of two pro-inflammatory cytokines (IL-6 and TNF-α) were observed, indicating that an HFD could induce low-grade systemic inflammation. However, B2 intervention significantly reduced the levels of circulating LPS and LBP and decreased the serum IL-6 while increasing IL-10, suggesting that B2 could ameliorate the inflammation in HFD-fed mice. Therefore, the modification of inflammation by B2 may also be responsible for its efficacy in preventing IR.

Insulin resistance is related to the adipocyte hormones (leptin and adiponectin) that mediate insulin-sensitizing effects through the activation of adenosine monophosphate dependent kinase (AMPK), PPARγ, and some other signaling pathways (25). In the present study, B2 intervention decreased the mRNA expression of *SHP* and *PPAR*γ. It is reported that the overexpression of SHP and PPARγ in HFD-fed mice constrained insulin-stimulated glucose uptake and damaged the insulin signaling pathway (18, 31). Additionally, SHP plays an essential role in the feedback of bile acid biosynthesis (32). Interestingly, B2 treatment increased the hepatic bile acids. B2 may regulate the bile acids resulting in improved insulin sensitivity through SHP and PPARγ pathways.

Obesity and hyperglycemia induced by an HFD were closely associated with the gut microbiota dysbiosis, which has been confirmed in this study. In addition, recent researches have also demonstrated that probiotics intervention exhibited promising roles, such as antiobesity and insulin sensitivity by restoring the balance of the intestine microorganisms in murine models (24, 33). In this study, B2 intervention decreased the α-diversity in comparison with the HFD group, indicating that B2 characteristically altered the gut bacterial community. Gut bacteria-derived bile salt hydrolase (BSH) is a major enzyme that catalyzes the “gateway” reaction in the biotransformation of conjugated BAs to produce deconjugated BAs (34). Furthermore, B2 treatment varied intestinal microflora with an enrichment of the intestine bacteria (health-promoting) with BSH-containing phyla (*Firmicutes* and *Actinobacteria*). Additionally, the *L. plantarum* was only found in HFD+B2 groups (Supplementary Figure 1), which suggested that *L. plantarum* B2 can be colonized in the gut. Moreover, *L. plantarum* B2 itself has high BSH activity. BAs are closely correlated to the glycolipids metabolism (35). Thus, B2 may exert its beneficial effects through the modulation of the gut microbiota on BAs.

In line with the previous findings, an HFD can increase the relative abundance of *Oscillospira* and *norank_f_Desulfovibrionaceae* in feces (36, 37). The B2 intervention significantly overturned the increased *Oscillospira* and *norank_f_Desulfovibrionaceae*, which were found to be more abundant in hyperglycemic and hyperlipidemic individuals in comparison with normal subjects (37, 38). *F16* was also identified as novel superior microbiota in probiotics intervention groups, which was consistent with a previous study (39). Based on LEfSe analysis, the genera *Oscillospira, Lactococcus*, and *Mucispirillum*, which were the characteristic flora of the HFD group, were found to be positively correlated to the LPS, suggesting that these bacteria may play a pro-inflammatory role in the development of obesity and IR (37, 40). It was also speculated whether the disorder of glycolipid metabolism could be modified by the beneficial gut microbes by preventing inflammation. One reason may be that helpful microbes could promote the bioconversion of fermentable carbohydrates into SCFAs (41). In this case, the SCFAs-producing bacteria (*Bifidobacteriaceae* and *Helicobacteraceae*) were increased in the B2 group. Consequently, mice fed on B2 produced more SCFAs, especially acetate. A study demonstrated that SCFAs largely ameliorated the glucose metabolism and IR disorders by reducing the production of LPS (42). These findings referred that B2 was a potent alternative in improving the beneficial gut bacteria in HFD-fed mice. Conclusively, B2 intervention could possibly provide a robust modulation of the gut microbiota, which possesses a promising antidiabetic potential.

Study findings revealed that B2 intervention showed promising potential in preventing HFD-induced insulin resistance was associated with the improvement of the gut microbiota and inhibition of LPS synthesis. B2 intervention significantly increased fecal BA excretion by increasing BSH comprising gut bacteria. Moreover, B2 intervention also promoted fecal SCFAs production. Based on the study findings, it is suggested that B2 intervention possibly used in preventing/improving hyperglycemia linked with alteration of the gut microbiota and their metabolites, including BAs and SCFAs. However, antidiabetic roles of the axis “gut microbiota - bile acids - liver” of B2 intervention in HFD-fed mice need to be confirmed by further investigations.

## Data Availability Statement

The datasets presented in this study can be found in online repositories. The names of the repository/repositories and accession number(s) can be found in the article/Supplementary Material.

## Ethics Statement

The animal study was reviewed and approved by Animal Ethical Committee of Zhejiang Chinese Medical University (approval no. ZSLL-2019-10947).

## Author Contributions

FF and RG conceived and designed research. HZ conducted experiments. JW and HZ contributed new reagents or analytical tools. HZ and FF analyzed data. HZ and A wrote the manuscript. A, MH, FF, and RG reviewed and edited the manuscript. All authors have read and approved the manuscript.

## Funding

This research was supported by Zhejiang Provincial Key Research and Development Program (Grant No. 2021C04032).

## Conflict of Interest

The authors declare that the research was conducted in the absence of any commercial or financial relationships that could be construed as a potential conflict of interest.

## Publisher's Note

All claims expressed in this article are solely those of the authors and do not necessarily represent those of their affiliated organizations, or those of the publisher, the editors and the reviewers. Any product that may be evaluated in this article, or claim that may be made by its manufacturer, is not guaranteed or endorsed by the publisher.
