# Supplementary material for: Lactobacillus plantarum ZJUFB2 Prevents High Fat Diet-Induced Insulin Resistance in Association With Modulation of the Gut Microbiota
Source: Front Nutr. 2021 Oct 14;8:754222. doi: 10.3389/fnut.2021.754222 (PMC8604096; doi:10.3389/fnut.2021.754222)
Supplement: Supplementary file 2 [file Table_2.docx]

**Table S1.** The ingredients and energy densities of the diets.

| **Ingredients (g / 100 g diet)** | **NCD** | **HFD** |
| --- | --- | --- |
| Casein | 18.96 | 23.31 |
| L-Cystine | 0.28 | 0.35 |
| Corn Starch | 29.86 | 8.48 |
| Maltodextrin | 3.32 | 11.65 |
| Sucrose | 33.17 | 20.14 |
| Cellulose | 4.74 | 5.83 |
| Soybean Oil | 2.37 | 2.91 |
| Lard | 1.90 | 20.68 |
| Mineral Mix | 2.68 | 3.31 |
| Potassium Citrate, 1 H2O | 1.56 | 1.92 |
| Vitamin Mix | 0.95 | 1.16 |
| Choline Bitartrate | 0.19 | 0.23 |
| **Calories supplementation (kcal %)** |  |  |
| Proteins | 20 | 20 |
| Carbohydrates | 70 | 35 |
| Fats | 10 | 45 |
| **Total calories (kcal / 100 g diet)** | 385 | 473 |

**Table S2.** The forward primer (F) and reverse primer (R) sequence used for qPCR amplification.

|  | **Primer name** | **Sequence (5’ to 3’)** |
| --- | --- | --- |
| 1 | β-actin-F | GTGCTATGTTGCTCTAGACTTCG |
| 2 | β-actin-R | ATGCCACAGGATTCCATACC |
| 3 | PPARγ-F | GCATTTCTGCTCCACACTATGA |
| 4 | PPARγ-R | TCGCACTTTGGTATTCTTGG |
| 5 | PPARα-F | GTCCTCAGTGCTTCCAGAGG |
| 6 | PPARα-R | GGTCACCTACGAGTGGCATT |
| 7 | SHP-F | GGAGTATGCGTACCTGAAG |
| 8 | SHP-R | TGCCTGGAATGTTCTTGAG |
| 9 | FAS-F | TGATGTGGAACACAGCAAG |
| 10 | FAS-R | GGCTGTGGTGACTCTTAGTGATAA |
| 11 | SREBP-1c-F | CCAGCGGCTGCCTTCACACA |
| 12 | SREBP-1c-R | CCAGCCGAAAAGCGAGGCCA |
| 13 | CYP7A1-F | TGGGCATCTCAAGCAAACAC |
| 14 | CYP7A1-R | TCATTGCTTCAGGGCTCCTG |


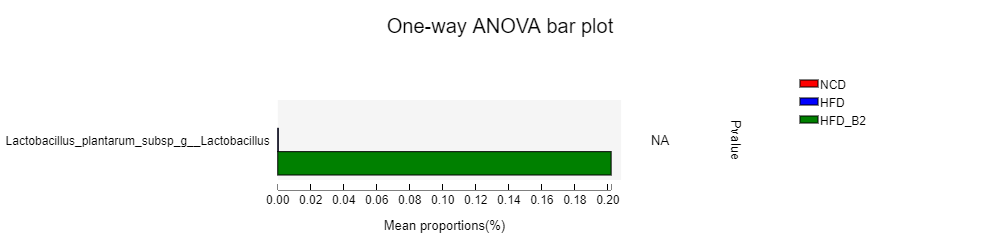


**Figure S1. Effects of *Lactobacillus plantarum* B2 on gut microbiota at *Lactobacillus plantarum* species in HFD-fed mice.**
